# Refugia or reservoir? Feral goats and their role in the maintenance and circulation of benzimidazole-resistant gastrointestinal nematodes on shared pastures

**DOI:** 10.1017/S0031182023000380

**Published:** 2023-07

**Authors:** Emily Kate Francis, Jan Šlapeta

**Affiliations:** 1 Sydney School of Veterinary Science, Faculty of Science, The University of Sydney, New South Wales 2006, Australia; 2 The University of Sydney Institute for Infectious Diseases, New South Wales 2006, Australia

**Keywords:** Benzimidazole resistance, cryptic species, isotype-1 *β*-tubulin, ITS-2 rDNA, livestock, nemabiome metabarcoding, *Teladorsagia circumcincta*, wild ruminants

## Abstract

Gastrointestinal nematodes threaten the productivity of grazing livestock and anthelmintic resistance has emerged globally. It is broadly understood that wild ruminants living in sympatry with livestock act as a positive source of *refugia* for anthelmintic-susceptible nematodes. However, they might also act as *reservoirs* of anthelmintic-resistant nematodes, contributing to the spread of anthelmintic resistance at a regional scale. Here, we sampled managed sheep and cattle together with feral goats within the same property in New South Wales, Australia. Internal transcribed spacer 2 (ITS-2) nemabiome metabarcoding identified 12 gastrointestinal nematodes (*Cooperia oncophora*, *Cooperia punctata*, *Haemonchus contortus*, *Haemonchus placei*, *Nematodirus spathiger*, *Ostertagia ostertagi*, *Teladorsagia circumcincta*, *Oesophagostomum radiatum*, *Oesophagostomum venulosum*, *Trichostrongylus axei*, *Trichostrongylus colubriformis* and *Trichostrongylus rugatus*). Isotype-1 *β*-tubulin metabarcoding targeting benzimidazole resistance polymorphisms identified 6 of these nematode species (*C. oncophora*, *C. punctata*, *H. contortus*, *H. placei*, *O. ostertagi* and *T. circumcincta*), with the remaining 3 genera unable to be identified to the species level (*Nematodirus*, *Oesophagostomum*, *Trichostrongylus*). Both ITS-2 and *β*-tubulin metabarcoding showed the presence of a cryptic species of *T. circumcincta*, known from domestic goats in France. Of the gastrointestinal nematodes detected *via β*-tubulin metabarcoding, *H. contortus*, *T. circumcincta*, *Nematodirus* and *Trichostrongylus* exhibited the presence of at least one resistance genotype. We found that generalist gastrointestinal nematodes in untreated feral goats had a similarly high frequency of the benzimidazole-resistant F200Y polymorphism as those nematodes in sheep and cattle. This suggests cross-transmission and maintenance of the resistant genotype within the wild ruminant population, affirming that wild ruminants should be considered potential reservoirs of anthelmintic resistance.

## Graphical abstract

**Figure:**
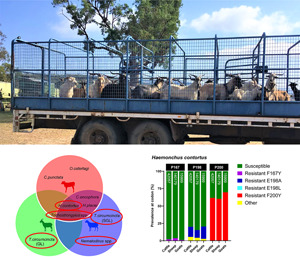

## Introduction

Gastrointestinal nematodes (GINs) are ubiquitous among domestic and wild ruminants, and many are generalist parasites capable of infecting multiple host species. GINs have a complex multiphase lifecycle which involves a parasitic phase inside the host, and a free-living stage in the environment (Zajac, 2006; Taylor *et al*., 2007). This means trophic transmission (i.e. ingestion of infective larvae) can occur between hosts in the absence of direct contact (Anderson, 2000). Under favourable environmental conditions, infective larvae (L3) can persist in the environment for 3–6 months (Barger *et al*., 1972), providing a large window of opportunity for transmission into sympatric host species who share pasture and/or water resources (host-switching) (Walker and Morgan, 2014). When a generalist GIN switches hosts, it could be carrying with it either the susceptible or anthelmintic-resistant genotype. Despite anthelmintic resistance (AR) being widespread within the livestock industry, the presence of resistance mutations conferring AR in wild ruminants is rarely explored (Brown *et al*., 2022). As such, the degree to which wild ruminants interfere with GIN management efforts in livestock remains mostly unknown. It has been theorized that wild ruminants, having never been treated with anthelmintic drugs, might act as an important source of *refugia* for anthelmintic-susceptible GINs, slowing the development of AR in livestock by diluting-out the resistant genotype (Van Wyk, 2001; Csivincsik *et al*., 2017; Hodgkinson *et al*., 2019). On the contrary, free-roaming wild ruminants could also propagate, maintain and circulate anthelmintic-resistant generalist GINs, exacerbating AR on a regional scale (Brown *et al*., 2022).

Feral goats (*Capra hircus*) are recognized worldwide as an agricultural pest and were first introduced to Australia during European settlement in the late 1700s (Parkes *et al*., 1996). They have since exploited a range of landscapes, but are most populous in arid and semi-arid regions which are also used for pastoral farming of sheep and cattle. With a population of approximately 2.3 million, and an individual home range of up to 460 km^2^ (DPI, 2022), feral goats are an ideal model for studying the transfer of generalist GINs and AR at the livestock–wildlife interface. Despite this, the majority of studies on host–nematode interactions and control of GIN species have been carried out in sheep, with GINs in goats being severely underrepresented in genetic databases, emphasizing the need for caprine-specific studies (Hoste *et al*., 2010). Our knowledge of the parasitic helminths of feral goats in Australia is outdated (McKenzie *et al*., 1979; Beveridge *et al*., 1987); relying only on the morphological identification of adult nematodes during routine abattoir surveillance. The most recent of these surveys was conducted over 3 decades ago and essentially all the feral goat GINs that were reported were said to be found in sheep (except for *Camelostrongylus mentulatus* and *Nematodirella dromedarii*, which are camelid GINs) (Spratt and Beveridge, 2019). To the best of our knowledge, no studies in Australia have investigated the GINs of feral goats in sympatry with sheep and cattle, and no studies have incorporated the surveillance of AR. This is likely due to the limitations of the available diagnostics at the time the research was conducted.

In recent years, modern non-invasive molecular tools have replaced outdated diagnostic techniques like morphological identification, individual polymerase chain reaction (PCR) assays and *ex-situ* experimental studies, which lack the efficiency and scalability required for robust GIN monitoring or longitudinal AR surveys. Deep amplicon nemabiome metabarcoding of the internal transcribed spacer 2 (ITS-2) region has made it possible to identify multiple GIN species in pooled larval samples (Avramenko *et al*., 2015), or directly from feces (Francis and Šlapeta, 2022), and has been deployed in North America and Europe to screen GINs in wild ruminants (Barone *et al*., 2020; Beaumelle *et al*., 2021). Furthermore, nemabiome metabarcoding of the isotype-1 *β*-tubulin gene has been used to identify the 3 main non-synonymous single nucleotide polymorphisms (SNPs), F167Y (TTC>TAC), E198A (GAA>GCA) and F200Y (TTC>TAC), which are associated with benzimidazole resistance in livestock (Avramenko *et al*., 2019; Sargison *et al*., 2019; Evans *et al*., 2021). This technique is yet to be applied to wild ruminants, with benzimidazole resistance only ever being revealed in wild deer through allele-specific PCR of adult abomasal *Haemonchus contortus* (Chintoan-Uta *et al*., 2014; Nagy *et al*., 2017).

In this study, we investigated the transmission of GINs between extensively reared sheep and cattle, and feral goats. Firstly, using ITS-2 nemabiome metabarcoding, we identified the extent to which generalist GIN species overlap in the 3 sympatric hosts, and then using *β*-tubulin metabarcoding, we determined whether benzimidazole-resistant nematodes are present in the feral goat population. Given the feral goats have never been treated with anthelmintics, this would most likely indicate GIN cross-transmission from livestock and elucidate the possibility that feral goats are capable of maintaining and circulating AR between livestock farms.

## Materials and methods

### Study area and sampling

**Figure 1.**
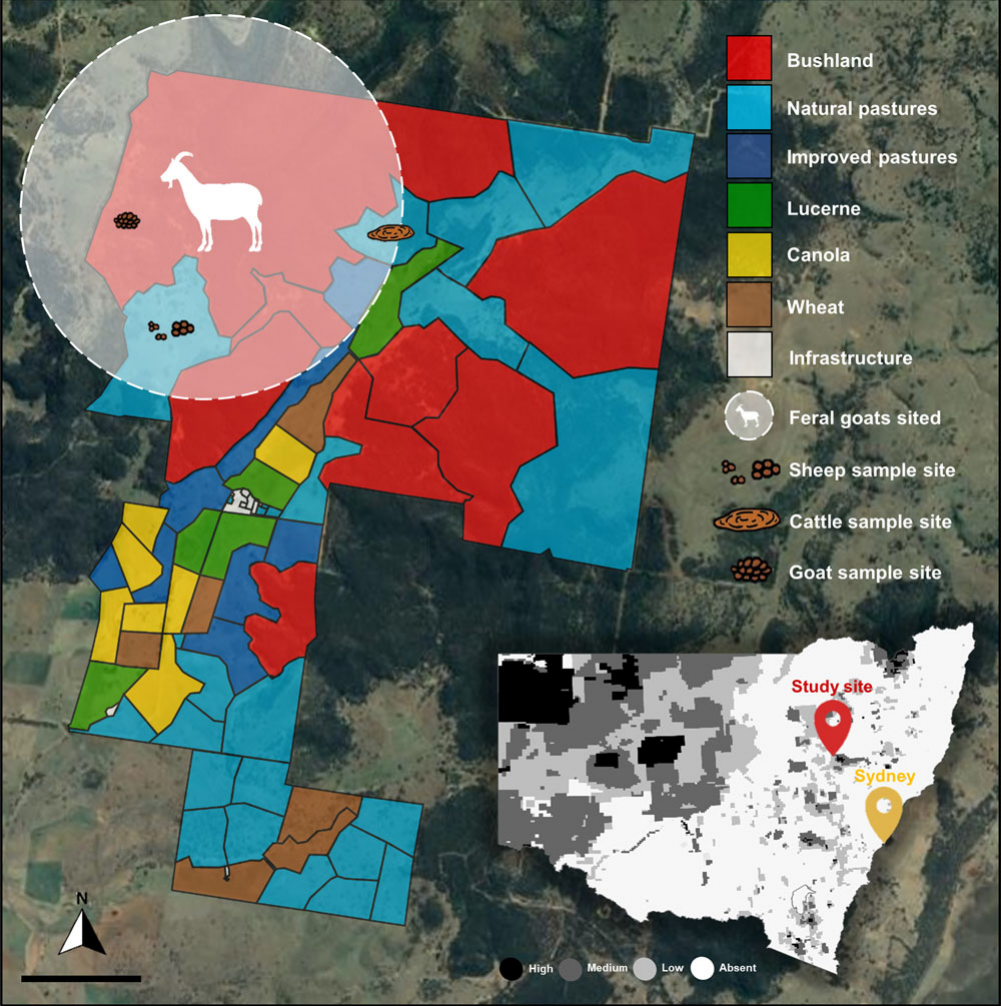
Land usage and location of feral goats at the study site, ‘Miangulliah’ Dunedoo (31°54’S, 149°34’E), a 2830 ha extensively managed mixed farming system located in central west New South Wales. The map and classification of land usage in each paddock at the time of sample collection was obtained from farm management software (AgriWebb, Australia). The superimposed map shows the 2016 distribution and abundance of feral goats in New South Wales recorded by the Department of Primary Industries (NSW DPI). The study site is located within a region classified as having a medium-to-high density of feral goats. Native and improved perennial pastures are used for rotationally grazing sheep and cattle, as well as cropping land (wheat, canola and lucerne). Feral goats are the most abundant wild ruminant on the farm and are regularly sited within the area circled. Freshly voided fecal samples were collected in January 2021, from sympatric sheep, cattle and feral goats, at the sampling sites indicated.

**Figure 2.**
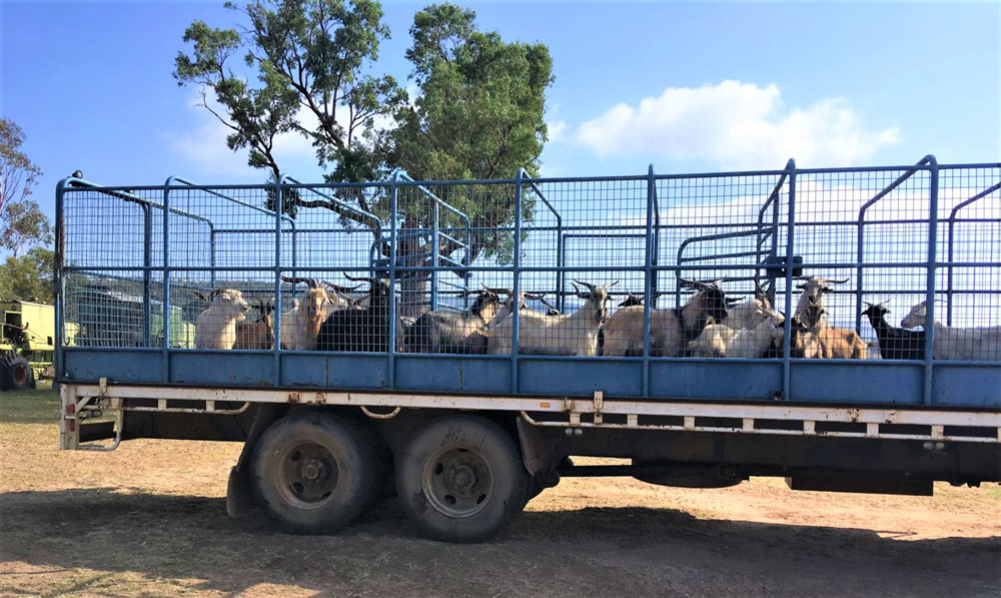
Feral goats loaded on the back of a truck at ‘Miangulliah’, Dunedoo, ready for sale. When the opportunity arises, for economic benefit goats are occasionally mustered into transportable yards and sold to the abattoir. They are not domesticated, nor have they ever been treated with anthelmintics.

Samples were collected from ‘Miangulliah’ Dunedoo (31°54’S, 149°34’E), a 2830 ha extensively managed mixed farming system located in central west New South Wales, 350 km north-west of Sydney (Fig. 1). The farm experiences a semi-arid to subtropical climate and encompasses a valley which is composed of both native and improved perennial pastures (for grazing sheep and cattle), and cropping land (mostly wheat, oats, canola and lucerne). Surrounding the valley is a dense native box-gum woodland which is home to an estimated population of 400 feral goats. Feral goats are the most abundant wild ruminant on the farm and are often observed in mobs of ~30 along the creek or grazing nearby pastures. For economic benefits, occasionally when the opportunity arises, feral goats are mustered into traps and sent straight to the abattoir (Fig. 2). These goats have never been domesticated nor have they ever been treated with anthelmintics. Sheep (~3350 head) and cattle (~600 head) are rotationally grazed according to age and/or production targets, except for lambing when sheep are set stocked at low densities (2.5–6 head/ha depending on feed availability). Sheep and cattle are strategically drenched at critical time-points (i.e. pre-lambing, weaning) or when significant GIN burdens are suspected. Anthelmintics used include a combination of macrocyclic lactones, benzimidazoles and levamisole.

Fecal samples (*N* = 10) were collected from sheep, cattle and feral goats (total *N* = 30) on the 27 January 2021. After observing a mob of ~30 feral goats grazing in a paddock at the fringe of the woodlands, individual feces were directly harvested off the ground by prospecting the grazed area. Only samples visually and physically identified as being fresh (i.e. glossy and warm to the touch) were collected. Feces that were less than 2 m apart and identical in size/shape were ignored to minimize the possibility of collecting multiple samples from the same individual. No sheep were grazing the paddock at this time. The same environmental sampling technique was used for the sheep (ewes) and cattle (weaners) in adjacent paddocks. The ewes and weaners were last treated with anthelmintics at the start of November 2020, using Trifecta® triple active oral drench (abamectin, levamisole, oxfendazole) (Coopers® Animal Health, Australia). Fecal samples were collected in 70 mL sterile specimen jars, sealed and placed in an esky with cool packs for direct transportation to the parasitology laboratory at the University of Sydney, Australia. Samples were refrigerated overnight at 4°C, and processed the following day.

### Fecal egg counts, coproculture and L3 gDNA purification

The number of GIN eggs per gram of feces (EPG) was counted for all individual samples using the highly sensitive Mini-FLOTAC technique (Department of Veterinary Medicine and Animal Productions, University of Naples Federico II, Italy). Mini-FLOTACs were performed according to the manufacturer's instructions for ruminants (Cringoli *et al*., 2017), and were examined under a light microscope (Olympus BX41, Australia) at 40× magnification. The analytical sensitivity and multiplication factor was 5 EPG. Individual larval cultures were set-up by hand-mixing a slurry of the remaining feces with water [~20 g feces: 20 mL double-distilled water (ddH_2_O)] and vermiculite (5 g), followed by incubation for 7 days at 27°C (Lyndal-Murphy, 1987). Culture jars were humidified with ddH_2_O twice during incubation to ensure the feces retained sufficient moisture for larval development. Third-stage infective larvae (L3) were harvested as described in Francis *et al*. (2020) and individual aliquots of 1000 L3 were stored overnight at 4°C. L3 samples were centrifuged at 1000 ***g*** for 2 min and total genomic DNA was extracted from the resulting pellets using the Monarch Genomic DNA Purification Kit (New England Biolabs, Australia). Extractions were conducted according to the manufacturer's instructions for tissue lysis and a ‘blank’ sample (ddH_2_O) was included as a negative control to monitor contamination. All larval extracts were eluted into a final volume of 100 *μ*L pre-heated (60°C) elution buffer (10 mm Tris-HCl, pH = 9.0, 0.1 mm ethylenediaminetetraacetic acid), and stored at −20°C.

### qPCR amplification of ITS-2 rDNA and isotype-1 *β*-tubulin gene

Stage-1 quantitative PCRs (qPCRs) for both the ITS-2 rDNA and isotype-1 *β*-tubulin nemabiome assays were performed on all gDNA lysates in-house (Francis *et al*., 2020). Illumina adapter primers were previously described and validated (Avramenko *et al*., 2015; Avramenko *et al*., 2019). For both assays, equal concentrations of the forward and reverse primers were mixed to create single forward and reverse primer mixes with final concentrations of 10 *μ*M. qPCRs were performed at a final volume of 20 *μ*L, containing SensiFAST™SYBR® No-ROX mix (Meridian Bioscience, Australia), forward and reverse primer mixes and 2 *μ*L of template DNA. Both qPCR assays were run on a CFX96 Real-Time PCR Detection System (BioRad, Australia) under the following 3-step conditions: initial denaturation at 95°C for 3 min, followed by 25–35* cycles of 95°C for 5s, 60°C for 15s, 72°C for 15s and a final melt curve analysis (*cycles were carefully selected to ensure all reactions were still in their exponential phase and had not plateaued). All qPCR runs included a no template control (ddH_2_O) and the first 10 reactions were run on a 1% agarose gel stained with 0.1% GelRed (Biotium, USA) and visualized under a UV transilluminator to ensure amplicons were the required size.

### Illumina MiSeq nemabiome metabarcoding and data handling

Individual qPCR amplicons were submitted for Illumina next-generation sequencing (NGS) at the Ramaciotti Centre for Genomics, University of New South Wales, Sydney, Australia. Here, stage-2 indexing PCRs, library quantification, normalization, pooling and denaturing were performed according to Illumina's 16S Metagenomic Sequencing Library Preparation (Illumina Inc., USA). Sequencing runs were undertaken on an Illumina MiSeq platform version 2.0 with 2 × 250 bp and de-multiplexed FASTQ files were automatically generated. ITS-2 rDNA and isotype-1 *β*-tubulin sequences were analysed separately using the DADA2 bioinformatic pipeline (Callahan *et al*., 2016) outlined at www.nemabiome.ca., performed on Artemis HPC (Sydney Informatics Hub, The University of Sydney). In summary, primers were removed from all forward and reverse reads using Cutadapt, quality profiles were plotted to determine suitable filtering parameters, error profiles were plotted and denoised, and paired-end reads were merged to reconstruct the full amplicon sequence variant (ASV) prior to chimaera removal. For the isotype-1 *β*-tubulin sequences, goat samples had to be re-analysed separately to sheep and cattle in DADA2, as the justConcatenate = TRUE function was required to successfully merge lengthy reads [particularly for *T. circumcincta* goat line (GL)].

### ITS-2 rDNA species assignments and phylogenetic analysis

ITS-2 ASVs were assigned to the species level using an in-house bespoke database of local trichostrongylid nematode ITS-2 rDNA reference sequences curated from previously published datasets derived from the GINs of livestock in New South Wales, Australia (Francis *et al*., 2020; Francis and Šlapeta, 2022) (LabArchives: https://dx.doi.org/10.25833/h6a4-be50). The database was imported into DADA2 using the ‘assignTaxonomy’ function, which assigns ASVs to the nearest match using the Bayesian classifier method, with a bootstrapping approach to assess the confidence assignment at each taxonomic level (Wang *et al*., 2007). To confirm species assignments, ASVs were also visualized in CLC Main Workbench 21.0.4 (CLC Bio, Qiagen, Australia), and any ASVs that were not assigned to the species level using the ‘assignTaxonomy’ function were aligned to the BLASTN GenBank database (NCBI, USA), only considering matches from reputable, published scientific papers. ASVs not unambiguously identified at this stage, or that accounted for <0.1% of total reads across all samples, were considered spurious and removed. The phylogenetic relationship between the final ASVs was visualized using Kimura-2 (K2) distance Minimum Evolution (ME) trees with bootstrap support (500 replicates) constructed in MEGA X (version 11.0.8) (Kumar *et al*., 2018). Species compositions were calculated by dividing the total reads assigned to each species by the total number of reads per sample. All species with a prevalence of <0.5% in a single sample were discarded as artificial or contaminating species. The inverse-Simpson index was calculated to measure *α* diversity, and to ensure an equal comparison, all samples were subsampled to 2000 reads.

### Identification of SNPs at isotype-1 *β*-tubulin codons 167, 186 and 200

Isotype-1 *β-*tubulin ASVs were assigned to the species level using the published database outlined in Avramenko *et al*. (2019). Species were assigned using the same bioinformatic workflow as described above for ITS-2 rDNA, with the exception that instead of discarding all ASVs that did not return a positive species identification, those that were able to be identified to the genus level using phylogenetic analysis on CLC Main Workbench were kept due to the lack of available isotype-1 *β-*tubulin reference sequences for some of the GINs detected *via* ITS-2 nemabiome metabarcoding (*Nematodirus spathiger*, *Oesophagostomum venulosum*, *Oesophagostomum radiatum* and *Trichostrongylus rugatus*). Final ASVs were visualized in CLC Main Workbench and aligned to a well-known *H. contortus β-*tubulin reference sequence (X67489) (Kwa *et al*., 1993) which was labelled at positions F167, E198 and F200. ASVs were manually screened at these positions for resistant genotypes: F167Y (TTC>T**A**C), E198A (GAA>G**C**A), E198L (GAA>**TT**A or **CT**A) and F200Y (TTC>T**A**C). The percentage of susceptible and resistant genotypes at each codon position was calculated by dividing the total reads assigned to each genotype by the total number of reads per species/genus. GINs with <1000 reads in any given host were not considered for SNP analysis for that particular host. Resistant genotypes with a prevalence of <1% at any codon were also considered spurious and excluded.

### Data visualization and accessibility

All ITS-2 rDNA and isotype-1 *β*-tubulin nemabiome numerical data were analysed in Microsoft Excel (version 2207) and visualized in GraphPad Prism (version 8.4.3). Circular stacked bar charts were created in R studio (version 1.4.1717) using ggplot2 (https://ggplot2.tidyverse.org). Raw FASTQ sequence data were deposited at SRA NCBI BioProject: PRJNA936713. Local ITS-2 trichostrongylid nematode databases and associated supplementary data are available *via* LabArchives (https://dx.doi.org/10.25833/h6a4-be50).

## Results

### Major livestock GINs identified in feral goats, along with cryptic *Teladorsagia circumcincta* species

The mean FEC of sheep (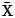 = 1329 EPG, range: 200–2920 EPG) and goat (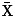 = 1442 EPG, range: 245–3145 EPG) feces were similar, whilst cattle feces were expectedly lower (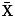 = 194 EPG, range: 40–365 EPG). All 30/30 fecal samples were successfully cultured into >2000 L3 and total genomic DNA was isolated from aliquots of ~1000. Ten L3 isolates from each host were used for ITS-2 nemabiome metabarcoding, and were all stage-1 qPCR positive with a mean Cq of 22.27 (s.d. ±4.31). Using the Illumina MiSeq and DADA2 pipeline, we obtained a final total of 458 895 ITS-2 paired-end reads (an average of 15 296 reads per sample) that were processed into 53 unique ASVs. All 53/53 ASVs were successfully assigned to the species level based on close identity (>96%) to the bespoke local or GenBank ITS-2 reference databases (Table 1). A total of 12 trichostrongylid nematode species were identified: *Cooperia oncophora*, *Cooperia punctata*, *H. contortus*, *Haemonchus placei*, *N. spathiger*, *Ostertagia ostertagi*, *Teladorsagia circumcincta*, *O. radiatum*, *O. venulosum*, *Trichostrongylus axei*, *Trichostrongylus colubriformis* and *T. rugatus.*

**Table 1.** Molecular identity of internal transcribed spacer 2 (ITS-2) Illumina next-generation amplicon sequence variants (ASV)

Species	ASV (DADA2)	Reference/s	Identity (%)	Host/s
*Cooperia oncophora*	ASV16^1^, ASV31^2^, ASV34^3^, ASV51^4^	USYD009^1^, USYD011^2^, USYD010^3^, USYD012^4^	100%^1,2,3,4^	Cattle, sheep
*Cooperia punctata*	ASV44	USYD019	100%	Cattle
*Haemonchus contortus*	ASV1, ASV2, ASV7, ASV15, ASV19, ASV20, ASV21, ASV26, ASV33, ASV37, ASV38, ASV40, ASV49, ASV53	LS997563	96.09–100%	Cattle, sheep, goat
*Haemonchus placei*	ASV3^1^, ASV4^2^, ASV50^1^, ASV52^2^	USYD004^1^, USYD008^2^	99.57–100%^1,2^	Cattle, sheep
*Nematodirus spathiger*	ASV22, ASV24, ASV45, ASV47	KC998747	99.22–100%	Sheep, goat
*Ostertagia ostertagi*	ASV6^1^, ASV12^2^, ASV39^3^, ASV48^2^	USYD021^1^, USYD022^2^, USYD023^3^	100%^1,3^ 99.58–100%^2^	Cattle
*Teladorsagia circumcincta*	ASV5^1^, ASV10^1^, ASV13^1^, ASV18^1^, ASV27^2^, ASV30^1^, ASV36^3^, ASV42^4^	AF351782^1^, USYD024^2^, USYD026^3^, USYD025^4^	99.21–100%^1^ 100%^2,3,4^	Sheep, goat
*Oesophagostomum radiatum*	ASV14^1^, ASV23^2^, ASV32^1^	USYD007^1^, USYD006^2^	98.66–100%^1^ 100%^2^	Cattle
*Oesophagostomum venulosum*	ASV8, ASV11, ASV25, ASV43	Y10790	98.44–99.61%	Cattle, sheep, goat
*Trichostrongylus axei*	ASV17^1^, ASV41^2^	USYD033^1^, USYD034^2^	100%^1,2^	Cattle, sheep, goat
*Trichostrongylus colubriformis*	ASV29	USYD032	100%	Goat
*Trichostrongylus rugatus*	ASV9^1^, ASV28^2^, ASV35^3^, ASV46^2^	USYD029^1^, USYD030^2^, USYD031^3^	100%^1,3^ 99.58–100%^2^	Goat

Numerical superscripts indicate which ASVs were assigned to each reference and the associated identity (%).

**Figure 3.**
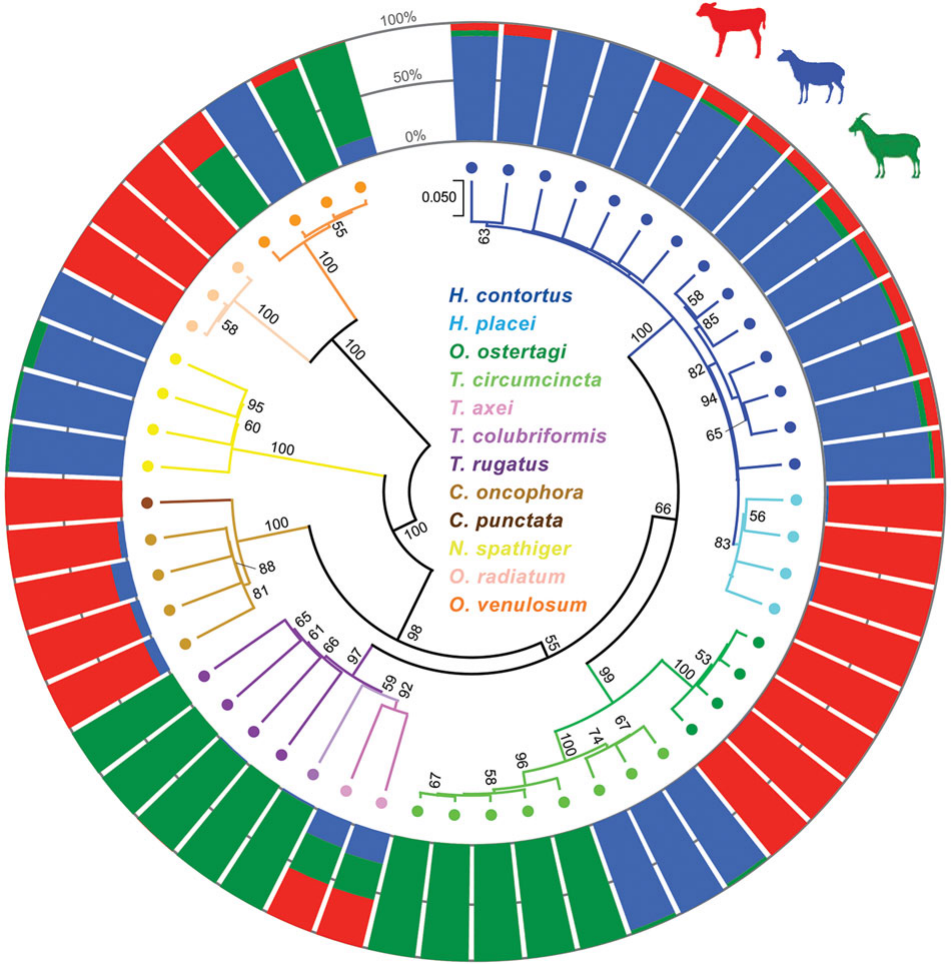
Phylogenetic relationship of internal transcribed spacer 2 (ITS-2) amplicon sequence variants (ASVs) from livestock and sympatric feral goat fecal samples. A total of 53 unique ITS-2 ASVs were obtained from larval nemabiome metabarcoding *via* the Illumina MiSeq platform and DADA2 pipeline. The phylogenetic tree was computed using the Minimum Evolution and Kimura-2 distance matrix on MEGA X version 11.0.8 (https://www.megasoftware.net). Bootstrap support values (>50%) from 500 replicates are shown at the branches. Circular-stacked bar charts were created in R studio (version 1.4.1717) using ggplot2 (https://ggplot2.tidyverse.org) and show individual ASVs as a percentage of total Illumina reads that belong to each host (cattle: red, sheep: blue, goats: green).

**Figure 4.**
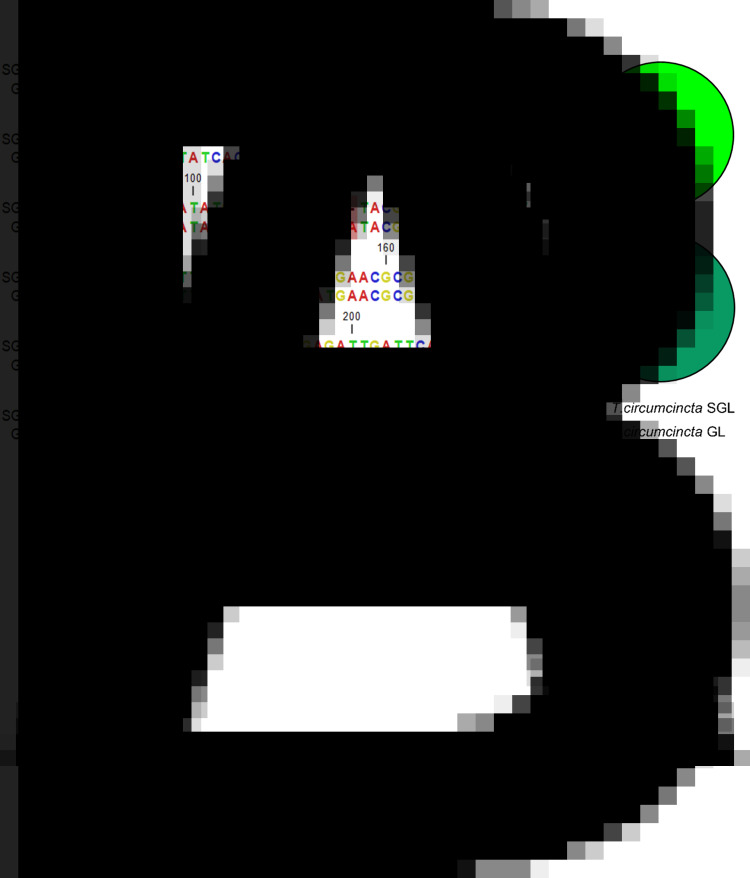
Comparison of *Teladorsagia circumcincta* internal transcribed spacer 2 (ITS-2) amplicon sequence variants (ASVs) which formed 2 separate monophyletic groups, known as the sheep and goat line (SGL) and goat line (GL). (A) Alignment of the consensus sequences of each line using CLC Main Workbench 21.0.4 (CLC Bio) confirms a cumulative 6 bp insertion (shaded red) in the GL, along with 4 point mutations (shaded grey) at positions 124, 128, 132 and 167. (B) Pie charts of the percentage of total Illumina reads of *T. circumcincta* SGL and GL in sheep and feral goats reveal preferential host species.

Phylogenetic analysis of the ASVs revealed both interspecies and intraspecies variation between the 12 trichostrongylid nematode species identified (Fig. 3). Of particular interest was *T. circumcincta*, which formed 2 distinct monophyletic groups with preferential host species, as similar findings were reported in a French Meunier population of goats (Leignel *et al*., 2002). An alignment of the consensus sequences of each monophyletic group exposed a cumulative 6 bp insertion (2 bp: P27–28, 1 bp: P114, 3 bp: P121–123) in one of the groups, along with 4 other point mutations (P124, P128, P132 and P167) (Fig. 4A). Pairwise comparison of each ASV identified sequence differences of 4.31–6.27% between the groups. For perspective, this dissimilarity was of a larger magnitude than what was observed between the 3 *Trichostrongylus* species detected in this study (2.35–3.92%). Of the total reads assigned to the species *T. circumcincta* in sheep, 100% belonged to the group of ASVs that did not contain the insertion, whilst in goats >99% (99.38%) contained the insertion and <1% (0.62%) didn't (Fig. 4B). Accordingly, the group containing the insertion was referred to as the goat line (GL) and the latter was referred to as the sheep and goat line (SGL).

**Figure 5.**
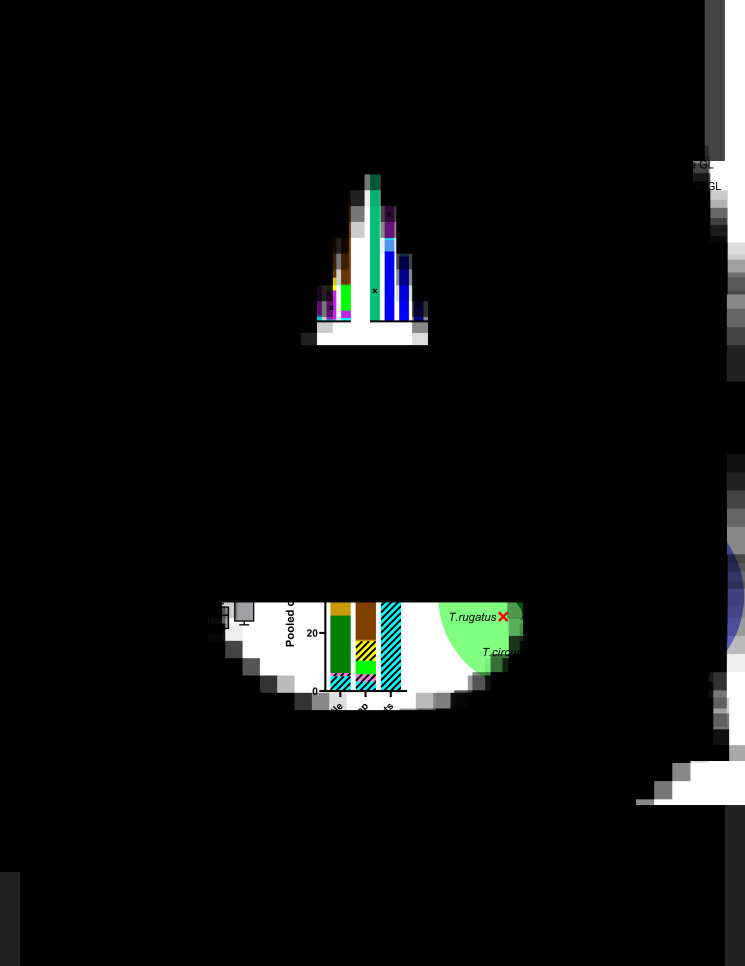
Gastrointestinal nematode compositions detected in sheep, cattle and feral goats on shared pastures. Individual (A) and pooled (B) larval nemabiome compositions were obtained *via* internal transcribed spacer 2 deep amplicon sequencing of 10 fecal samples from each host, with individual fecal egg counts (as indicated by the ‘X’ on the bars) calculated using the Mini-FLOTAC technique (Department of Veterinary Medicine and Animal Productions, University of Naples Federico II, Italy). (C) Box and whisker plots compare the *α* diversity of individual samples between sympatric hosts, as measured by the inverse-Simpson index. (D) Pooled nemabiome compositions *via* isotype-1 *β*-tubulin deep amplicon sequencing were also visualized (genera not able to be identified to the species level are shown in diagonal lines). (E) Venn diagram showing the degree of sharing between gastrointestinal nematodes and hosts and the species that were unable to be detected to the species level *via β*-tubulin nemabiome metabarcoding (indicated by a red cross).

Comparison of the ITS-2 nemabiomes of the 10 cattle, sheep and goats revealed the extent to which GINs are shared between sympatric hosts and differences in GIN diversity (Fig. 5A). Overall, *H. placei* was the most prevalent GIN species in cattle accounting for 43.52% of the pooled species composition, followed by *O. ostertagi* (26.22%) and *H. contortus* (13.42%), and small contributions of *O. radiatum*, *C. oncophora*, *T. axei*, *O. venulosum* and *C. punctata* (7.84, 5.59, 1.67, 1.25 and 0.49%, respectively) (Fig. 5B). Sheep were predominately infected by *H. contortus* (87.47%) with minimal contributions of *N. spathiger*, *O. venulosum*, *T. circumcincta* SGL, *T. axei*, *H. placei* and *C. oncophora* (4.59, 3.61, 2.37, 0.87, 0.61 and 0.48%, respectively). The most prevalent GIN species in goats was the goat-specific *T. circumcincta* GL (50.55%), *O. venulosum* (24.95%) and *T. rugatus* (16.06%), followed by *H. contortus*, *T. colubriformis*, *T. axei*, *T. circumcincta* SGL and *N. spathiger* (4.68, 1.63, 1.57, 0.32 and 0.25%, respectively). The *α* diversity of GINs was highest in the goats, with a mean inverse-Simpson index of 2.21 (s.d. ±0.99), followed by cattle (1.83, s.d. ±0.79) and sheep (1.23, s.d. ±0.17) (Fig. 5C).

### *β*-Tubulin nemabiome metabarcoding confirms feral goats are potential reservoirs of benzimidazole resistance

Illumina NGS of the isotype-1 *β*-tubulin region was also conducted to surveil for SNPs associated with benzimidazole resistance in each host, but was limited by its inability to detect some GIN species to the level of ITS-2 nemabiome metabarcoding. We obtained a total of 325 925 *β*-tubulin paired-end reads for sheep and cattle (an average of 16 296 reads per sample), and 152 434 paired-end reads for goats (an average of 15 243 reads per sample) with <3% of total reads being removed as spurious sequences. Overall, 6 trichostrongylid nematode species: *C. oncophora*, *C. punctata*, *H. contortus*, *H. placei*, *O. ostertagi* and *T. circumcincta*, were identified based on close identity to the reference database (>96%). *Teladorsagia circumcincta* ASVs were classified as SGL or GL *via* alignment to published partial sequences of the reported *T. circumcincta* isotype-1 *β*-tubulin ‘type I’ and ‘type II’ variants found in French Meunier goats (Leignel *et al*., 2002). Some ASVs were unable to be unambiguously identified to the species-level, but were assigned to the genera: *Nematodirus*, *Oesophagostomum* and *Trichostrongylus*, based on phylogenetic analysis with the reference database. The proportions of *C. oncophora*, *C. punctata*, *H. contortus*, *H. placei* and *O. ostertagi* in *β*-tubulin pooled sheep and/or cattle were similar to ITS-2; however *N. spathiger*, *O. venulosum*, *O. radiatum* and *T. axei* were not successfully identified to the species level (Fig. 5D and E). *Trichostrongylus* spp. appeared to be over-represented in the pooled goats, *Oesophagostomum* spp. and *Nematodirus* spp. were not detected; however, the proportion of *T. circumcincta* GL appeared to be accurate when compared to the ITS-2 nemabiome.

**Figure 6.**
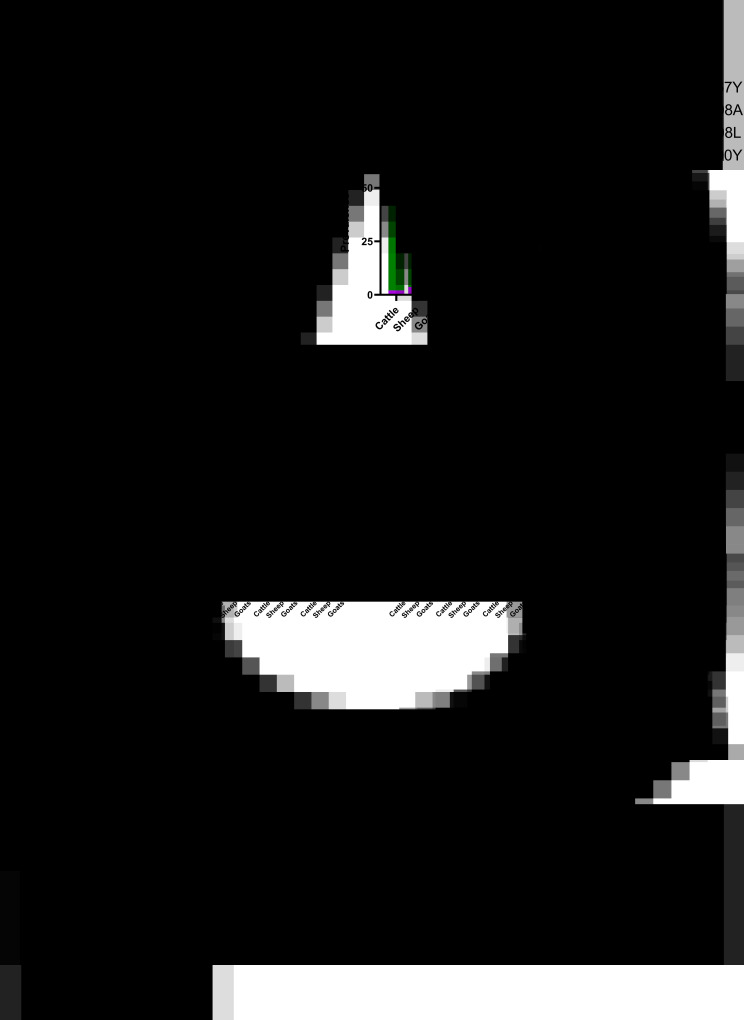
Comparison of the relative proportions of isotype-1 *β*-tubulin allele frequencies in sympatric livestock and feral goats for the 4 gastrointestinal nematode species/genera positive for benzimidazole resistance (A); *Haemonchus contortus* (B), *Trichostrongylus* spp. (C), *Nematodirus* spp. (D), *Teladorsagia circumcincta* SGL (E) and *Teladorsagia circumcincta* GL. Each bar includes a number that represents read depth. Susceptible alleles (F167, E198, F200) are shown in green, while previously described polymorphisms are displayed in various other colours (F167Y: purple, E198A: dark blue, E198L: light blue, F200Y: red). A novel non-synonymous polymorphism at codon 198 (E198G: GAA > G**G**A) is displayed in yellow and labelled as ‘other’. It is unknown whether E198G confers benzimidazole resistance. Hosts for which no isotype-1 *β*-tubulin sequences were detected for that species are indicated by N/A on each chart.

Screening of isotype-1 *β*-tubulin polymorphisms at codons 167, 198 and 200 revealed the presence of 4 known benzimidazole-resistant genotypes: F167Y (TTC>T**A**C), E198A (GAA>G**C**A), E198L (GAA>**TT**A) and F200Y (TTC>T**A**C), which were present at varying degrees amongst sympatric hosts. Of the trichostrongylid nematodes detected *via β*-tubulin metabarcoding, *H. contortus*, *T. circumcincta* SGL and GL, *Nematodirus* spp. and *Trichostrongylus* spp. exhibited the presence of 1 or more of these resistant genotypes, whilst *C. oncophora*, *C. punctata*, *H. placei*, *O. ostertagi* and *Oesophagostomum* spp. were entirely susceptible (Fig. 6A). Although there were differences in the absolute abundance of *H. contortus* between the hosts, which has already been described, the relative proportion of F200Y in *H. contortus* was similar across all hosts, but highest in goats (cattle: 61.78%, sheep: 60.46%, goats: 69.59%) (Fig. 6B). Cattle, sheep and goats were mostly susceptible at codon 198 (79.90, 84.57 and 79.50%, respectively), with small proportions of E198A (<17.73%) and a novel polymorphism E198G (GAA > G**G**A) (<5.46%), which was labelled ‘other’. E198G results in an amino acid change of glutamine to glycine (Glu > Gly) which has not yet been reported in any trichostrongylid nematode species, so its effects are unknown. All hosts were almost entirely susceptible at codon 167, with very minimal contributions of F167Y (<3.64%). A small frequency of F200Y was detected in *Trichostrongylus* spp. across all hosts (cattle: 7.71%, sheep: 18.06%, goats: 15.62%) (Fig. 6C), whilst *Nematodirus* spp. in sheep was almost entirely F200Y resistant (92.94%) (Fig. 6D). *Teladorsagia circumcincta* SGL in sheep exhibited resistant genotypes E198L (34.59%) and F200Y (65.41%) (Fig. 6E), while *T. circumcincta* GL appeared to be heterozygous at the P200 locus (Fig. 6F).

## Discussion

Deep amplicon NGS is re-defining the surveillance of complex nematode communities and their AR status in domesticated ruminants worldwide (Avramenko *et al*., 2019; Sargison *et al*., 2019; Evans *et al*., 2021). Similar studies in sympatric wild ruminants are lacking. Many GINs are presumed to be common to both domestic and wild ruminants (generalists), including some of the most pathogenic species with the highest incidences of AR (i.e. *H. contortus*) (Laca Megyesi *et al*., 2019). Despite this, the extent to which AR is being transferred to/or maintained in wild sympatric hosts has mostly been speculated (Brown *et al*., 2022). Our study presents the first investigation of benzimidazole resistance in wild ruminant parasites in Australia. Feral goats were the focus of this study due to their ubiquity across Australian pastoral zones, large home-range and similar physiology to domesticated sheep and cattle. Deep amplicon nemabiome metabarcoding of both the ITS-2 rDNA and isotype-1 *β*-tubulin regions provided molecular evidence of feral goats as a reservoir of AR generalist GINs in Australia, and identified the presence of a cryptic species of *T. circumcincta.*

Previous to this study, the most recent investigation into the parasitic fauna of feral goats in Australia (Beveridge *et al*., 1987) reported 18 GIN species (detected *via* morphological identification of adult nematodes), which is significantly more diverse than the 8 GINs we identified *via* ITS-2 nemabiome metabarcoding of L3. In our study, no ‘unknown’ ITS-2 ASVs were detected, with all ASVs being confidently assigned to a species in the reference databases. This decreased species diversity could suggest that over the past few decades, major generalist GINs have dominated the nemabiome of feral goats, at the expense of more rare specialist species; as was reported in populations of wild roe deer in Europe (Beaumelle *et al*., 2022). Contrary to this, the most prevalent GIN found in feral goats in our study was a cryptic species of *T. circumcincta* highly specialized to goats, only ever reported in a French Meunier population of domesticated goats (Gasnier and Cabaret, 1996; Leignel *et al*., 2002; Grillo *et al*., 2007). The generalist SGL morphotype was in much smaller proportions and could even be considered contamination due to the phenomenon of index hopping, which is a known limitation of NGS technologies (Costello *et al*., 2018). Nonetheless, the abundance of the GL in our study strongly supports the hypothesis that the current definition of *T. circumcincta* as a major generalist GIN of sheep and goats is masking a more complex population structure including the presence of multiple species with preferential hosts (Grillo *et al*., 2007).

It is well known that *H. contortus* and *H. placei* simultaneously infect cattle and small ruminants, particularly on communal pastures (Amarante *et al*., 1997; Achi *et al*., 2003). In tropical and sub-tropical regions of Australia and New Zealand, there is a dogma that the more pathogenic *H. contortus* is responsible for the majority of *Haemonchus* infections in young immune-naïve cattle (Waghorn *et al*., 2019), likely due to its enhanced fecundity and shorter prepatent period (Emery *et al*., 2016), and so a pre-weaning treatment of calves is often suggested in integrated sheep–cattle systems. In our study, the ITS-2 nemabiomes of weaners were dominated by the more specialized *H. placei*, with *H. contortus* only identified in 3 individuals. This is a significant finding for the region and suggests that pre-weaning treatments are not always justified, which could prolong the effectiveness of the available anthelmintics. The high proportions of *H. contortus* detected in sympatric sheep, at the cost of all other GIN species, were unsurprising due to significant amounts of summer rainfall at the time of sample collection (Besier *et al*., 2016). It was interesting that similar magnitudes of *H. contortus* were not seen in sympatric feral goats; perhaps due to differences in their regulatory response and feeding behaviours (Hoste *et al*., 2010).

Molecular techniques such as deep amplicon NGS are only as good as the database of DNA sequences that their primers were originally designed against, or that was available during species assignment. This was evident in the nemabiome metabarcoding of the isotype-1 *β*-tubulin region, which was unsuccessful in identifying 3 GIN genera to the species level (*Oesophagostomum*, *Nematodirus* and *Trichostrongylus*). Discrepancies in species assignments were most likely due to the lack of available reference sequences for the unidentified species (*N. spathiger*, *O. radiatum*, *O. venulosum*, *T. rugatus*), rather than variations at the *β*-tubulin region primer-binding sites. Initially, a large proportion of goat paired-end reads did not successfully merge without concatenation and it was suspected that this was due to significant variations in the size of isotype-1 *β*-tubulin DNA fragments of some GIN species, particularly the goat-specific *T. circumcincta* GL due to its substantially larger first intron in comparison to its morphotype; SGL (Leignel *et al*., 2002). This is the first time *β*-tubulin nemabiome metabarcoding has been applied to the GINs of a wild ruminant host so some limitations were expected. As more studies involving wild ruminants emerge and the available database of DNA sequences diversifies, the full capabilities of this technique will be realized.

Surveillance of the *β*-tubulin region for polymorphisms at codons 167, 198 and 200 confirmed that feral goats could play an important role in the maintenance and circulation of benzimidazole-resistant GINs on shared pastures. Three out of the 5 benzimidazole-resistant GINs detected on the farm successfully parasitized feral goats, albeit in varying proportions relative to sheep and cattle depending on the species examined. Whilst the absolute abundance of *H. contortus* was lowest in feral goats, the frequency of the F200Y genotype was similar, if not higher. High levels of BZ-resistant variants in an untreated host is almost certainly due to cross-transmission; however, this can only be inferred from our data and would require direct testing using longitudinal data from a controlled study. Goats are well recognized as ‘super-spreaders’ of GINs due to their high fecundity (Le Jambre and Royal, 1976), with fecal egg counts of up to 3145 EPG in this study. This, coupled with a large home-range, presents a significant concern for neighbouring farms with no history of AR. The exact magnitude of the impact feral goats have as reservoirs of benzimidazole-resistant GINs, along with their affinity to and maintenance of the resistant genotype, will be an important area of future research. In addition to this, a novel SNP at codon 198 (E198G) of *H. contortus* was detected in sheep, cattle and feral goats. The amino acid change of glutamine to glycine is not yet known to be associated with benzimidazole resistance in nematode species, but has been detected in benzimidazole-resistant fungi *Neurospora crassa* (Fujimura *et al*., 1992).

In conclusion, this study provides a proof-of-concept of the power of deep amplicon nemabiome metabarcoding to screen GINs and AR genotypes, and more importantly, the value of the information generated when applied to wild ruminants living in sympatry with sheep and cattle. A larger-scale, longitudinal study is warranted to confirm our findings in feral goats at a regional level and should incorporate other ubiquitous wildlife. Investigations into *T. circumcincta* as a species-complex should be reignited, as the cryptic GL is no longer just an isolated case in domesticated goats in France. Finally, wild ruminants should be treated as potential reservoirs of AR, and this should be considered when implementing GIN control strategies to delay the emergence of resistance.

## Data Availability

The data that support the findings of this study are openly available in LabArchives (https://dx.doi.org/10.25833/h6a4-be50) and raw FASTQ sequence data was deposited at SRA NCBI BioProject: PRJNA936713.
